# Validity of Mobility-Based Exposure Assessment of Air Pollution: A Comparative Analysis with Home-Based Exposure Assessment

**DOI:** 10.1021/acs.est.3c10867

**Published:** 2024-06-05

**Authors:** Lai Wei, David Donaire-Gonzalez, Marco Helbich, Erik van Nunen, Gerard Hoek, Roel C. H. Vermeulen

**Affiliations:** † Department of Human Geography and Spatial Planning, Utrecht University, 3584 CB Utrecht, The Netherlands; ‡ Institute for Risk Assessment Sciences, Utrecht University, 3584 CK Utrecht, The Netherlands; § Julius Centre for Health Sciences and Primary Care, University Medical Centre, Utrecht University, 3584 CK Utrecht, The Netherlands

**Keywords:** air pollution, GPS, personal exposure, PM_2.5_, black carbon, exposure assessment

## Abstract

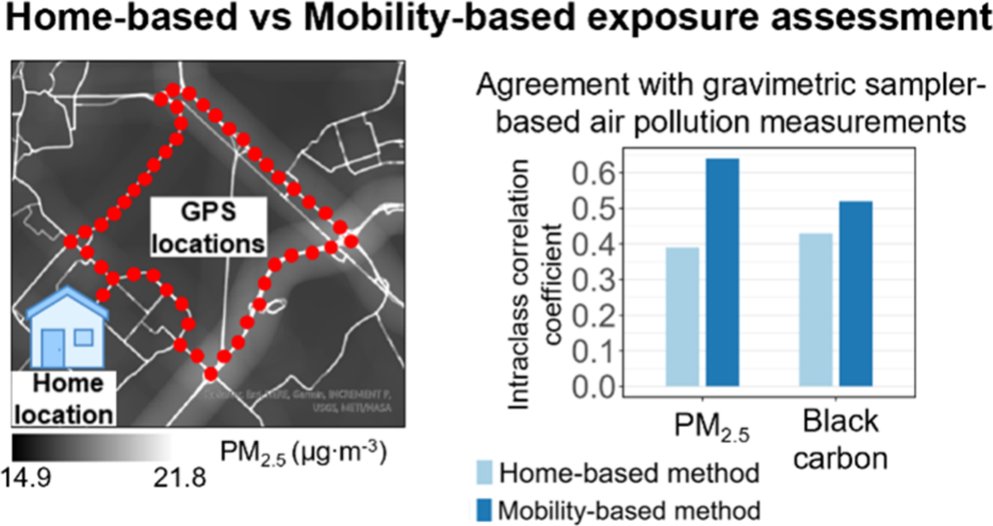

Air pollution exposure is typically assessed at the front door where people live in large-scale epidemiological studies, overlooking individuals’ daily mobility out-of-home. However, there is limited evidence that incorporating mobility data into personal air pollution assessment improves exposure assessment compared to home-based assessments. This study aimed to compare the agreement between mobility-based and home-based assessments with personal exposure measurements. We measured repeatedly particulate matter (PM_2.5_) and black carbon (BC) using a sample of 41 older adults in the Netherlands. In total, 104 valid 24 h average personal measurements were collected. Home-based exposures were estimated by combining participants’ home locations and temporal-adjusted air pollution maps. Mobility-based estimates of air pollution were computed based on smartphone-based tracking data, temporal-adjusted air pollution maps, indoor-outdoor penetration, and travel mode adjustment. Intraclass correlation coefficients (ICC) revealed that mobility-based estimates significantly improved agreement with personal measurements compared to home-based assessments. For PM_2.5_, agreement increased by 64% (ICC: 0.39–0.64), and for BC, it increased by 21% (ICC: 0.43–0.52). Our findings suggest that adjusting for indoor-outdoor pollutant ratios in mobility-based assessments can provide more valid estimates of air pollution than the commonly used home-based assessments, with no added value observed from travel mode adjustments.

## Abstract 2

Existing evidence on whether mobility-based air pollution assessment improves personal exposure assessment is insufficient. This study quantified the validity of the mobility-based approach with implications for improving exposure assessment.

## 1 Introduction

Air pollution is a major threat to global public health, attributable to about 6.7 million premature deaths yearly.^1^ Its adverse health effects, including cardiovascular diseases,^2,3^ mental health problems,^4^ and respiratory diseases,^5,6^ have been documented in many meta-analyses and reviews. However, the magnitudes of the estimated effect sizes were not always consistent. These inconsistencies could partly arise from how people’s exposure to air pollutants was estimated.

Air pollution assessments in large-scale epidemiological studies are typically conducted at the front door where people live^7−10^ by intersecting the address location with modeled air pollution concentration maps.^7,10,11^ However, these approaches likely lead to inaccuracies in air pollution exposure assessments because people do not spend all the time at home.^12^ People’s everyday life unfolds over multiple activity locations (e.g., workplace, recreation), and they experience different exposure concentrations along their day-to-day mobility.^13,14^ It is thus vital to assess air pollution exposures dynamically based on people’s mobility patterns.

Some attempts have been made to advance home-based air pollution exposure assessments to mobility-based assessments,^11,15,16^ including direct and indirect approaches. Direct approaches rely on personal portable air pollution monitors that directly measure air pollutant concentrations. However, multiple methodological and technical challenges have been brought forward; for example, the devices monitor only a selected number of pollutants and are limited by battery life and high costs, leading to limited measurements to estimate long-term exposures.^17^ These challenges prevent their use in large studies.

Indirect approaches assess air pollution concentration levels based on people’s spatiotemporal trajectories captured by global positioning system (GPS)-enabled devices (e.g., GPS-trackers, smartphones).^18−20^ By combining the GPS data with air pollution maps obtained through land use regressions (LUR), personal exposure to air pollution can be estimated. Due to the highly granular data on people’s whereabouts, diverse aspects of human behaviors and the microenvironment, such as dwelling time and travel model, can be taken into account to enhance the accuracy of exposure assessments.^16,21−24^ Compared to direct approaches, indirect assessments are more convenient and cheaper to apply to a large population and multiple exposures, and thus become more feasible and used in epidemiological studies.^11,14^

However, whether moving from home-based (static) to mobility-based (dynamic) approaches improves the validity of the exposure assessment remains largely unknown. Only a few studies compared the differences between the air pollution concentrations assessed statically and dynamically, and the results suggested pronounced differences between static and dynamic air pollution exposure assessments.^13,23,25−27^ For example, residential and dynamic estimated personal exposure to PM_2.5_ were significantly different and the difference could be up to 6%.^16,21,27,28^ However, personal exposure estimates were simulated and cannot capture the true exposure as measured with portable air pollution monitors. Due to the absence of personal exposure measurements, comparisons against ground-truth benchmark data were not made in past studies. Therefore, existing evidence is insufficient in terms of whether mobility-based approaches improve personal air pollution exposure assessment.

Identifying such an improvement for specific population groups (e.g., older adults) benefits the decision-making on adopting the suitable exposure assessment method in epidemiological studies. Older adults are subject to higher health risks and more vulnerable to the environment than other groups.^29^ Though the mobility-based method is considered to be suitable for identifying exposure to the environment in the general population, its applicability in older adults could be limited.^27^ Older adults usually present a different spatiotemporal mobility profile, characterized by less daily travel and smaller activity spaces, than younger adults.^30^ Given their daily activities are mostly conducted around the home locations, there are emerging concerns about whether using mobility-based assessment of older adults is really necessary.

To respond to these knowledge gaps, this study aimed to compare the agreement between home-based and mobility-based air pollution assessment methods and a ground-truth benchmark (e.g., direct approach-based personal measurements) using a sample of older adults. Our findings contribute to accurately quantifying the validity of the mobility-based method of air pollution assessment, and evaluating to what extent the mobility-based method improves the validity of exposure assessment compared to the standard home-based method.

## 2 Materials and Methods

### 2.1 Study Design

This study was conducted as a part of the EXPOsOMICS project.^31,32^ Participants were recruited mostly from cohort studies in the Netherlands.^31^ Inclusion criteria for eligible participants were: (1) aged 50–70 years; (2) in good health condition; (3) nonsmokers and no smoking in their home (i.e., not smoking combustible and electronic cigarettes); and (4) having a historic blood sample in an ongoing cohort study.^31^ For increasing the contrast in exposure, our objective was to enroll 50% of the participants living close to main roads (≥10,000 vehicles/day) and 50% at minor roads (<10,000 vehicles/day). In the end, we included 41 participants living in Amsterdam or Utrecht who were monitored for three nonconsecutive 24 h between March 2014 and February 2015. To minimize seasonal variation in personal mobility behavior and ambient air pollution,^33^ three monitoring activities for each participant took place in different seasons (spring/fall, summer, and winter). The Ethical Committee of the University Medical Center Utrecht approved the study protocol. Participants provided written informed consent before study inclusion.

### 2.2 Personal Exposure Monitoring

On the monitoring day, participants wore a belt equipped with a smartphone, a backpack fitted with a time-integrated gravimetric sampler of particulate matter with an aerodynamic diameter of 2.5 μm or less (PM_2.5_), and a time-resolved light absorbance device (aethalometer) measuring black carbon (BC).^34^ Participants’ mobilities were tracked with the preinstalled “ExpoApp” Android smartphone application, an integrated system monitoring multiple personal exposures that records geolocations every 10 s.^35^ During each monitoring session, participants also filled out a time-activity diary with a 5 min accuracy to record their locations (e.g., home, work, in-transit, or other), dwelling time (e.g., 15 min), and travels (e.g., car, bike, or tram). Additionally, home-based air pollution measurements at participants’ home locations were taken.

### 2.3 Air Pollution Measurement Methods and Data Processing

**Figure 1:**
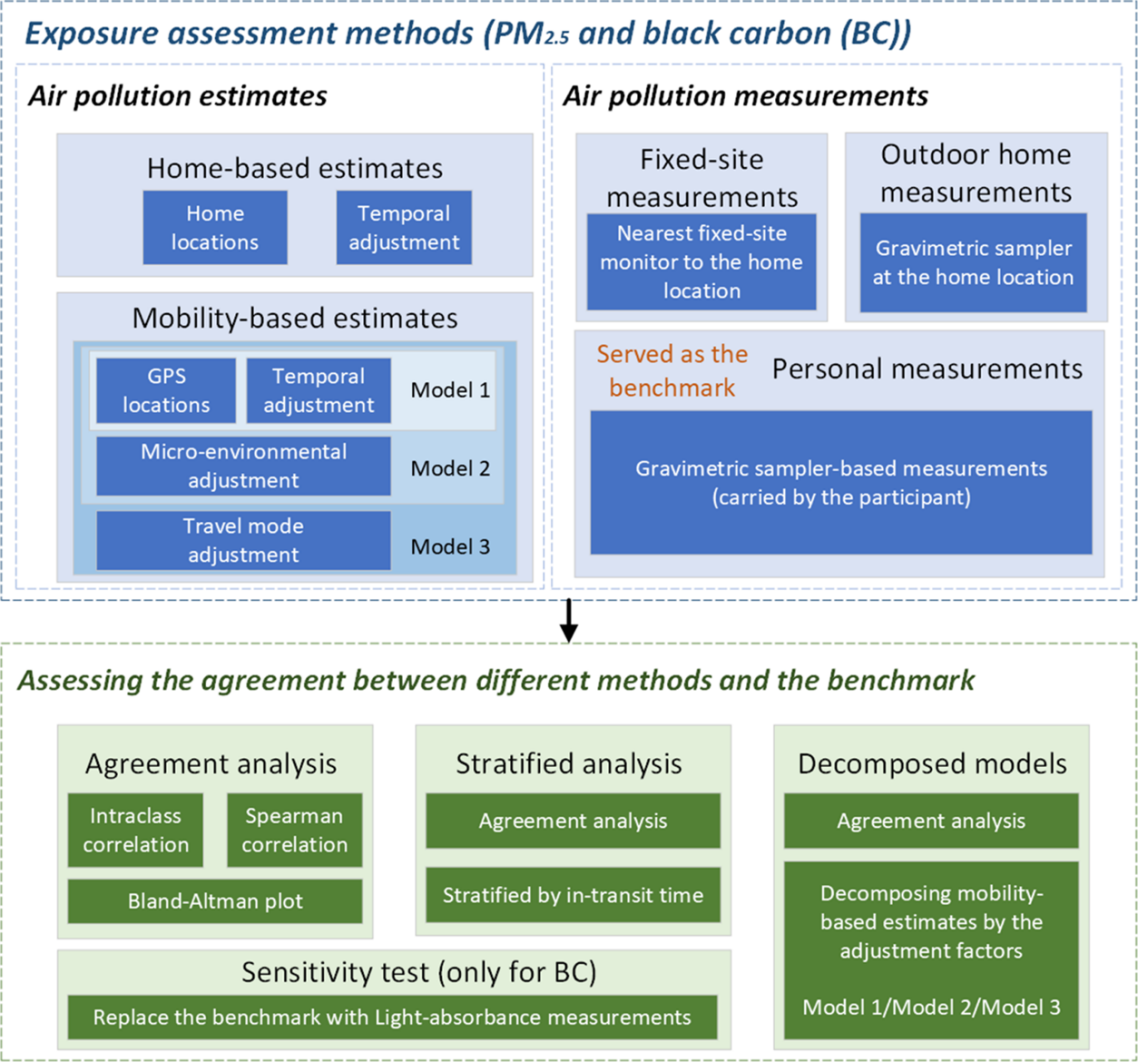
Workflow of this study.

We assessed participants’ exposure to PM_2.5_ and BC using standard measurement methods (Figure 1).

#### 2.3.1 Fixed-Site Measurements

PM_2.5_ and BC measurements at fixed monitoring sites (see Supporting Figure S1) were obtained from the Dutch National Air Quality Monitoring Network (https://www.luchtmeetnet.nl/). The measurements followed the European Air Quality Directive 2008/50/EC.^36^ Specifically, PM_2.5_ was measured with the MetOne beta-attenuation monitor and BC with a multi-angle absorption photometer (MAAP). We assigned air pollution levels from the geographically closest monitoring station to the participants’ home locations. Hourly monitored exposure levels over 24 h were averaged as the fixed-site measurement.

#### 2.3.2 Outdoor Home Measurements

Outdoor home measurements of PM_2.5_ and BC were collected by the gravimetrical samplers placed outside the participants’ main living room window. As reported elsewhere,^32^ PM_2.5_ and BC were sampled with the same 37 mm Teflon filters held in a cyclone (model GK2.05 SH, BGI, Inc., Waltham MA) (Supporting Figure S2) with an aerodynamic cut point of 2.5 μm and connected to a BGI/Mesa Labs A4004 pump working at 3.5 L/min. 24 h average PM_2.5_ concentrations were determined by the difference between pre- and postsampling filter weight using a microbalance of 1 μg accuracy (Model MT5, Mettler-Toledo International, Inc., Switzerland). The exposure level of BC was converted from PM_2.5_ reflectance, which was measured using a Smoke Stain Reflectometer (SSR) (Model 43D, Diffusion Systems Ltd., U.K.). Measurements were discarded if the sampling duration was less than 16 h and/or the end flow deviated more than 20% from the designed 3.5 L/min (2.8–4.2 L/min). Standardized operating procedures for collecting samples, analytical procedures, and quality control followed the ESCAPE procedures (manuals can be found at http://www.escapeproject.eu/manuals/).

#### 2.3.3 Personal Measurements

##### 2.3.3.1 Gravimetric Sampler-Based Measurements

Gravimetric sampler-based measurements of PM_2.5_ and BC were collected by the gravimetric samplers, the same as the devices for outdoor home measurements, carried by the participants. The details of devices, data collection, and cleaning procedures are described in Section 2.3.2. Our analysis considered the gravimetric sampler-based measurements of PM_2.5_ and BC as the benchmark. Unless otherwise stated, we refer to gravimetric sampler-based measurements as personal measurements.

##### 2.3.3.2 Light Absorbance Measurements

The concentration of BC was also measured using a MicroAeth monitor (model AE51, AethLabs, San Francisco, CA) with a 1 min temporal resolution (Supporting Figure S2). The validation report of the MicroAeth monitor can be found elsewhere.^37^ An Optimized Noise reduction Averaging (ONA) algorithm was applied to reduce measurement errors in the BC concentrations.^38^ Similar to gravimetric sampler-based measurements, only measurements with more than 16 h monitoring can be retained. This method was used as an alternative benchmark for BC in the sensitivity test.

Personal and outdoor home measurements were performed with the same instrumentation. Fixed-site measurements were performed with different instruments; thus, differences in the absolute level can be expected in comparisons of fixed-site measurements with personal and outdoor home measurements and estimated exposure (Section 2.4) based on another PM sampler. PM_2.5_ and BC from different instruments have been shown to be highly correlated.^21^ We did not colocate instruments to quantify differences for this study.

### 2.4 Air Pollution Estimate Methods

The exposures to PM_2.5_ and BC for each participant were also modeled using participants’ geolocations and air pollution maps (Figure 1).

#### 2.4.1 Land Use Regression Models for PM_2.5_ and BC

We used annual average PM_2.5_ and PM_2.5_ absorbance concentration maps for modeling personal exposures. The exposure to BC was estimated by PM_2.5_ absorbance as explained in Section 2.3, which is highly correlated with BC concentration.^21^ These maps were derived from ESCAPE LUR models predicted by traffic intensity, population, land use, and elevation.^39^ The models were derived from measurements made with the Harvard Impactor.^39^ The data referred to 2009 with a 5 m spatial resolution. The LUR model validation results are provided elsewhere.^40^

#### 2.4.2 Home-Based Estimates

The home-based PM_2.5_ and BC exposure estimates were determined by combining the LUR-based concentrations at the participants’ geocoded home locations and an hourly temporal adjustment factor. The adjustment factor extrapolated the air pollution concentration in 2009 to the concentration during the participants’ monitoring period. The factor was computed based on the time series measurement data and the LUR-based air pollution map at the closest fixed-site monitoring station.^21,39^ Specifically, for each monitoring station, we utilized the differences between the PM_2.5_ measurements during the monitoring period in an hour resolution and the PM_2.5_ concentrations at the location of the monitoring station based on the LUR model as the hourly temporal adjustment factor. Home-based PM_2.5_ estimates were determined by adding up the mean hourly temporal adjustment factor and the PM_2.5_ concentrations at the participants’ home locations in the LUR model.

For BC, we calculated the hourly temporal adjustment factor as the ratio between the hourly BC concentration measurement at the monitoring station and the concentration at the station’s location in the LUR-based air pollution map. The hourly home-based exposure was derived by multiplying this hourly temporal adjustment factor with the BC concentrations at the participants’ home locations in the LUR-based air pollution map. Subsequently, the home-based BC estimates were determined by averaging these hourly home-based exposures. The median temporal adjustment factors and their first and third quartiles over 24 h are shown in Supporting Figure S3.

#### 2.4.3 Mobility-Based Estimates

The mobility-based PM_2.5_ and BC estimates were computed using participants’ GPS-based tracking data in three steps. First, we extracted concentrations from the LUR-based air pollution surface at each GPS location and combined these values with a temporal adjustment factor. The temporal adjustment factor was computed based on air pollution measurements from the nearest monitoring station to each GPS point. For each GPS point, the temporal adjustment factor for PM_2.5_ was calculated as the difference between the measured PM_2.5_ concentration at the monitoring station at the recording time of the GPS point, and the PM_2.5_ concentration of the LUR model at the monitoring station’s location. We then obtained the PM_2.5_ exposure value for each GPS point by adding this temporal correction factor. Similarly, for BC, we utilized the ratio of BC measurements at the monitoring station to the LUR model value at the station’s location as the BC temporal adjustment factor. Subsequently, the BC exposure value for the GPS point was determined by multiplying the BC concentration by the temporal adjustment factor. Supporting Figure S2 shows the median, the first and third quartiles of temporal adjustment factors over 24 h.

Second, we extrapolated outdoor and indoor exposure using a microenvironmental factor. Each participant’s GPS tracking data were classified into four microenvironments (i.e., home, work, in-transit, and others) using a validated map-matching algorithm for travel-activity location classification.^35^ We reclassified home, work, and others as the indoor environment. Previous studies have shown that outdoor and indoor air pollution concentrations vary substantially.^41−43^ We therefore calculated indoor exposure concentrations from outdoor concentrations when participants were indoors. Hoek et al.^44^ derived different intercepts and slopes for relationships between 24 h average indoor and outdoor concentrations of PM_2.5_ and BC in Amsterdam. The microenvironmental factors were as follows:^44^ Indoor PM_2.5_ concentration = 4.7 + 0.39 × outdoor PM_2.5_ concentration; indoor BC concentration = 0.1 + 0.78 × outdoor BC concentration. Indoor sources of PM_2.5_ and BC were ignored.

Third, we applied a travel mode factor to extrapolate the exposure levels for different in-transit modes. Participants’ travel modes were acquired from the time-activity diary. Air pollution concentrations can be extrapolated between travel modes using the transport ratios with one type of travel mode as the constant of reference.^45^ Guided by a quantitative review study in European cities,^45^ the exposure levels for different travel modes were bike/walk (PM_2.5_ = 1.3; BC = 1.5), bus/walk (PM_2.5_ = 1.5; BC = 0.8), car/walk (PM_2.5_ = 1.4; BC = 2.9). We assumed that the LUR-based concentrations equal pedestrian-level exposure concentrations.^16^ We further assumed that the exposure concentrations were the same in buses, trams, metros, and trains, as we had no information about mode-specific concentration differences.^16^ The microenvironmental and travel model adjustment factors corrected exposure levels of PM_2.5_ and BC per GPS point. Averaged values from all GPS points were used as mobility-based estimates.

### 2.5 Statistical Analysis

Boxplots and raincloud plots summarized the estimated and measured PM_2.5_ and BC levels descriptively. Since exposure data did not follow normal distributions, Spearman correlation analysis was applied to quantify bivariate associations between different air pollution assessment methods. The intraclass correlation coefficient (ICC) quantified the agreement between personal measurements and other exposure assessment methods.^46^ Since each participant had multiple observations, we fitted linear mixed models with a random intercept for each participant to obtain the ICC.

Several indicators were adopted to compare the measurement error between different exposure assessment methods. First, we calculated the normalized mean bias factor (*B*_nmbf_) and normalized mean absolute error factor (*E*_nmaef_), as statistically robust measures of relative bias and error of a model.^47^ The level of agreement between methods was defined *a priori* as excellent if the ICC is >0.7, |*B*_nmbf_| < 0.25, and *E*_nmaef_ < 0.35, moderate if the ICC is between 0.5 and 0.7, or |*B*_nmbf_| ≥ 0.25, or *E*_nmaef_ ≥ 0.35.^47^

Second, we visualized the agreement between different exposure assessment methods and the benchmark with Bland–Altman plots, which plot the difference between two methods against the mean of two methods.^48^ Based on the Bland–Altman plots, we calculated the bias, and limits of agreement (i.e., mean bias ±1.96 standard deviation (SD) of the bias).

As sensitivity tests, we repeated the analyses after: (1) replacing the benchmark with light absorbance measurements for BC; (2) stratifying by participants’ median time in-transit to evaluate the impact of participants’ travel behavior on the validity of the mobility-based exposure assessment; and (3) decomposing the mobility-based method by each adjustment factor to evaluate their significance to the mobility-based exposure assessment. Three mobility-based models were compared: Model 1 was temporally adjusted, Model 2 was temporally and microenvironmentally adjusted, and Model 3 was fully adjusted including transportation mode. Unless stated otherwise, mobility-based estimates refer to Model 3. All analyses were conducted in R, version 4.1.2.^49^

## 3 Results

### 3.1 Study Population Characteristics and Exposure Distribution

Table 1 provides descriptive statistics of the study population. Study participants were, on average, aged 61.7 (SD ± 6.7) years, predominantly female (82.9%), highly educated (83%), and from urban areas (95.1%). They spent an average of 1.1 h (SD ± 0.7) in-transit, and 19 h (SD ± 3.8) at home. More than half (56.1%) of the subjects spent at least 30 min in green spaces.

**Table 1:** Characteristics of Study Participants (*N* = 41)

category	*n* (%)/mean (SD)
age (years)	61.7 (6.7)
sex:	
female	34 (82.9%)
male	7 (17.1%)
education level:	
any secondary school	1 (2.4%)
high school	6 (14.6%)
university or higher	34 (83.0%)
traffic volume at home location:	
low (<10,000 vehicles/day)	20 (48.8%)
high (≥10,000 vehicles/day)	21 (51.2%)
living in an urban area	39 (95.1%)
daily in-transit travel duration (hours)	1.1 (0.7)
daily duration at home (hours)	19 (3.8)
daily duration at other environment (hours)	2.2 (2.0)
time in green spaces, ≥30 min	23 (56.1%)
daily travel distance (km)	35.8 (47.9)

**Figure 2:**
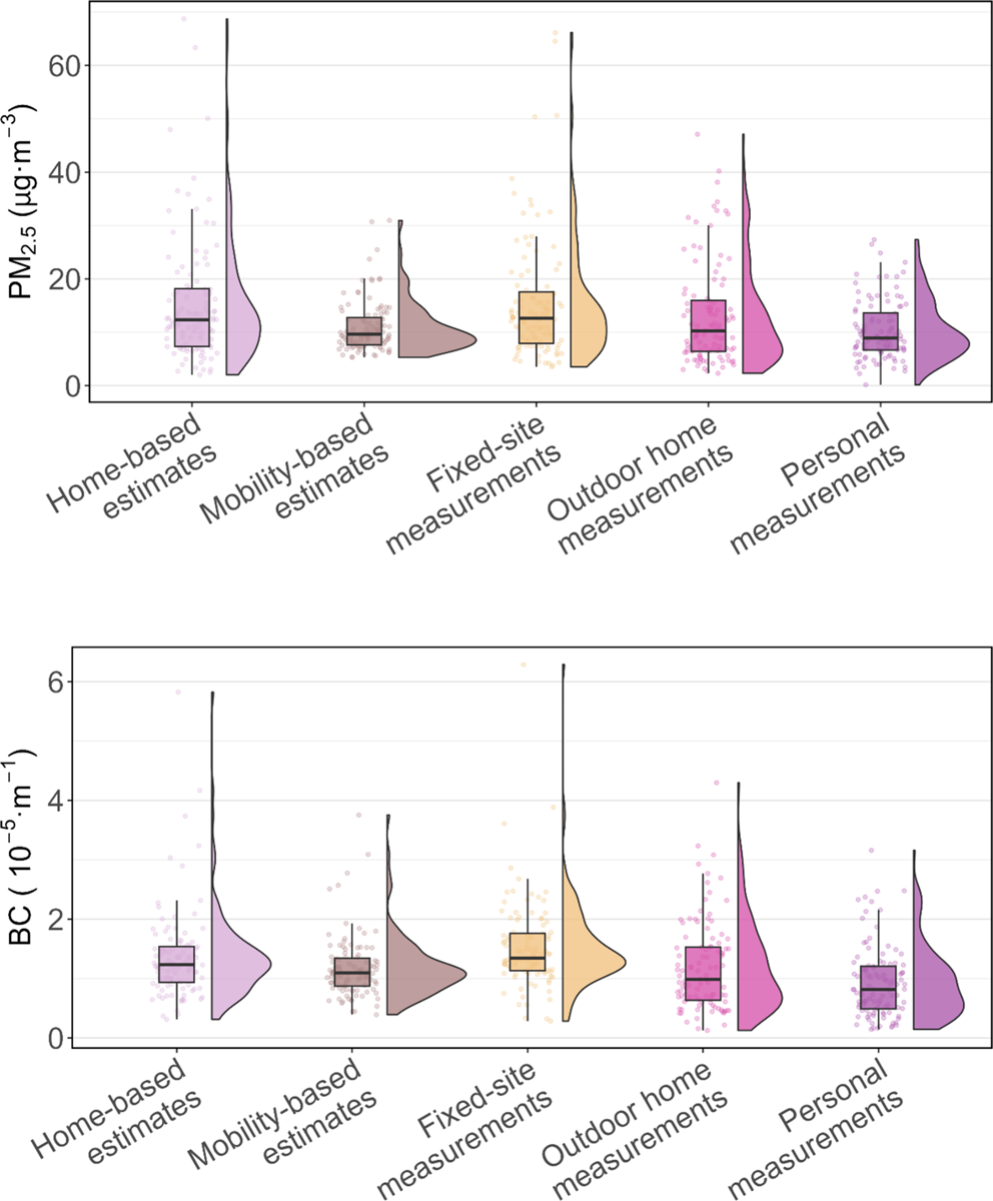
Distribution of the assessed PM_2.5_ and black carbon (BC) concentrations across different exposure assessment methods (*N* = 104). Mobility-based estimates refer to Model 3 which were adjusted for temporal, microenvironmental, and travel mode factors. Note that BC from fixed-site measurements is in the unit of μg·m^–3^; both BC and PM_2.5_ from fixed-site measurements were measured from different instruments than outdoor home and personal measurements.

There were 123 person-days of PM_2.5_ and BC measurements monitored. Due to partial measurement equipment failures, 104 person-days were available for outdoor home and personal measurements. There were 74 valid samples for real-time light absorbance measurements used for sensitivity analysis. Figure 2 and Supporting Table S1 show the PM_2.5_ and BC distribution across different methods. For both exposures, outdoor home measurements and home-based estimates showed considerably more variations and higher median exposure levels than personal measurements and mobility-based estimates. The median and interquartile range (IQR) of personal measurements were 8.92 (6.94) μg·m^–3^ and 0.82 (0.71) 10^–5^·m^–1^ for PM_2.5_ and BC, respectively (Supporting Table S1).

Supporting Figure S4 shows the correlation matrices across different methods. Personal measurements for both exposures were moderately correlated (0.63) with home-based and mobility-based estimates. Home-based and mobility-based estimates were highly correlated (0.97–0.99) for both exposures.

### 3.2 Agreement between Exposure Assessment Methods and the Benchmark

**Figure 3:**
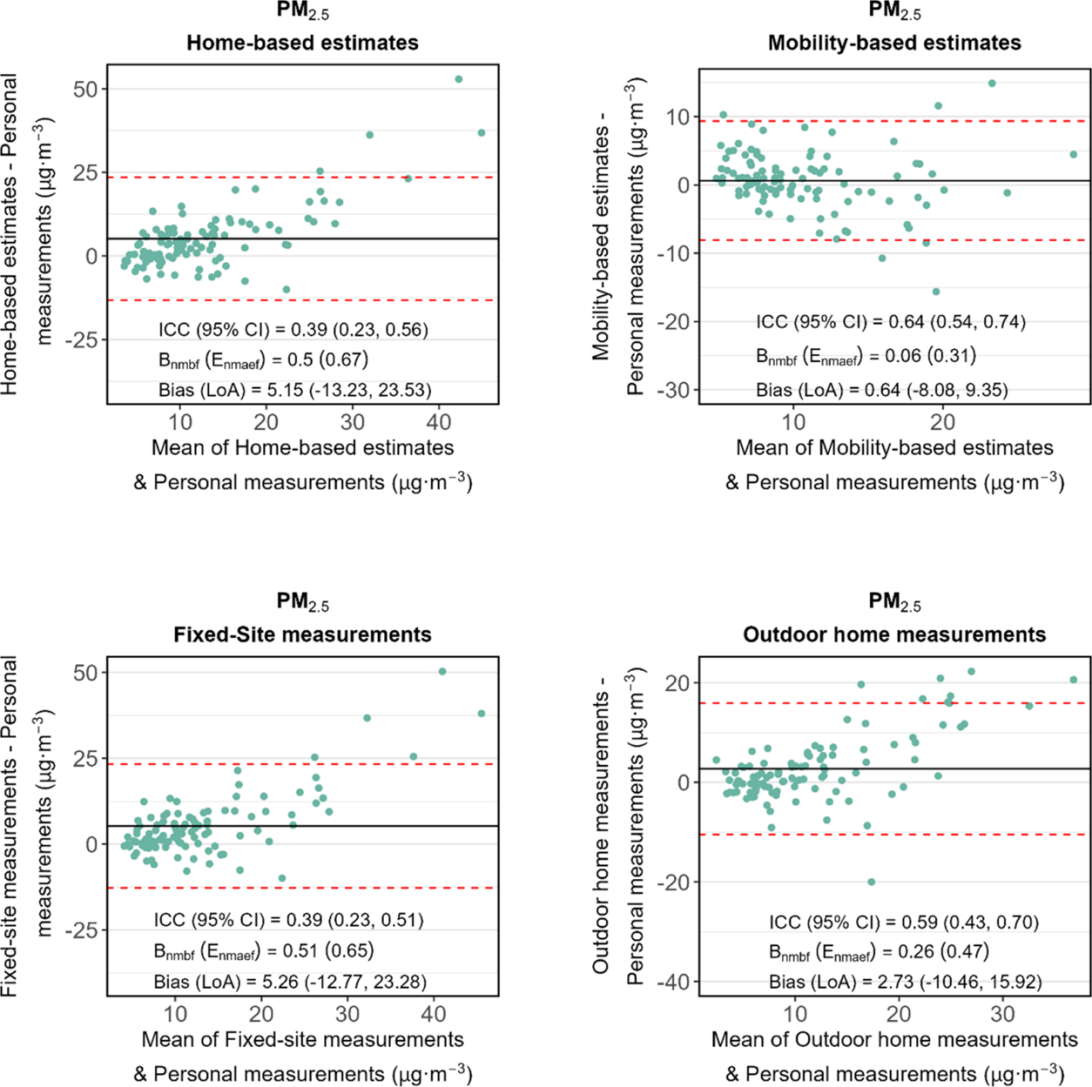
Bland–Altman plots of PM_2.5_ exposure levels between different exposure assessment methods and the benchmark (i.e., personal measurements). Limits of agreement (LoA; i.e., mean bias ±1.96 standard deviation of the bias) and bias are shown in red dashed lines and black solid lines, respectively. Mobility-based estimates refer to Model 3 which were adjusted for temporal, microenvironmental, and travel mode factors. ICC refers to the intraclass correlation coefficient.

**Figure 4:**
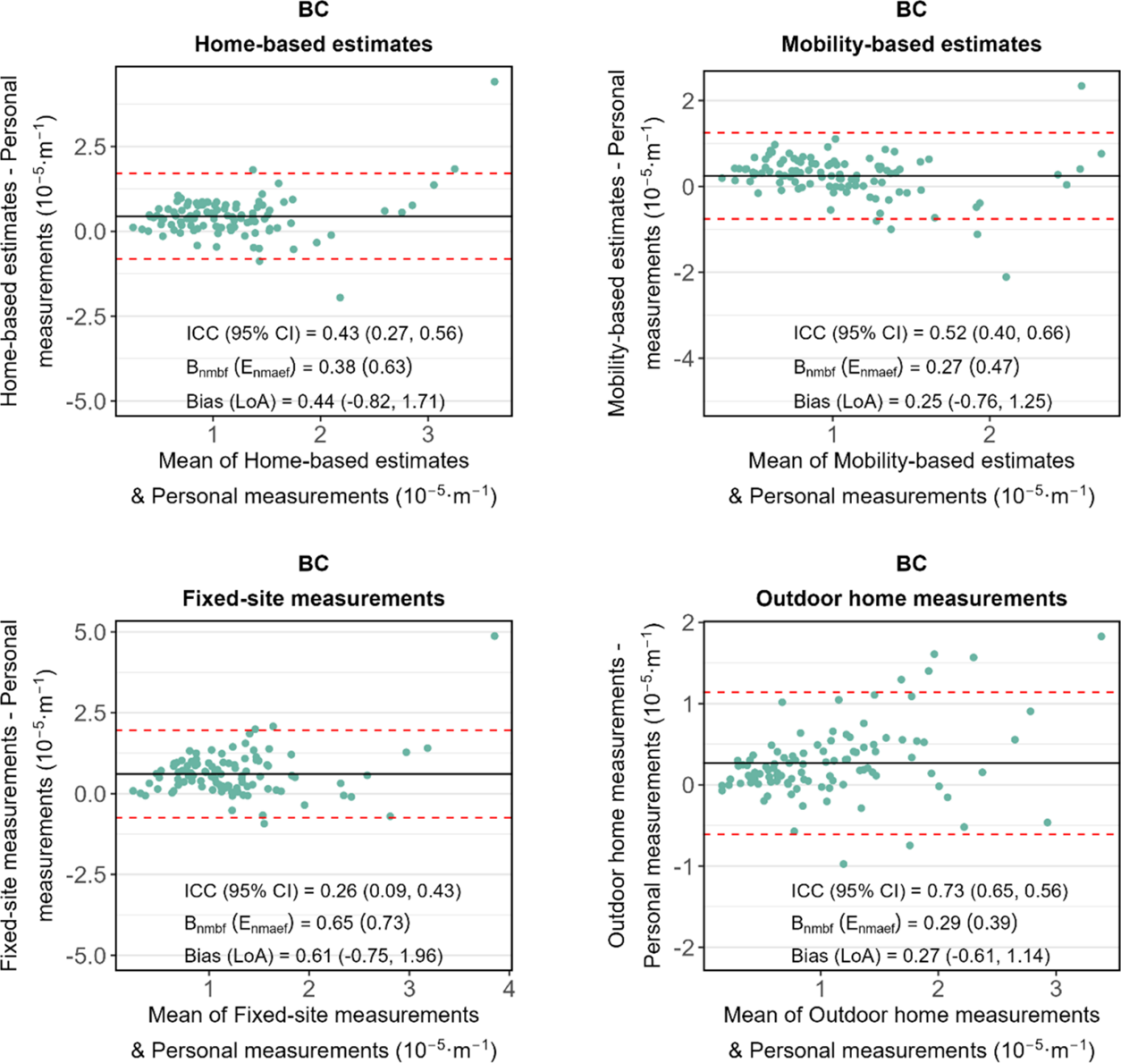
Bland–Altman plots of BC exposure levels between different exposure assessment methods and the benchmark (i.e., personal measurements). Limits of agreement (LoA; i.e., mean bias ±1.96 standard deviation of the bias) and bias are shown in red dashed lines and black solid lines, respectively. Mobility-based estimates refer to Model 3 which were adjusted for temporal, microenvironmental, and travel mode factors. ICC refers to the intraclass correlation coefficient.

Figure 3 displays the agreement between personal measurements (i.e., the benchmark) and other methods for PM_2.5_, and Figure 4 shows the results of BC. For PM_2.5_, no method achieved an excellent level of agreement. Mobility-based estimates and outdoor home measurements achieved moderate agreement; the former had a higher ICC. Compared to the ICC of home-based estimates, the ICC of mobility-based estimates increased from 0.39 to 0.64, improving about 64%. The bias was reduced from 5.15 to 0.64 μg·m^–3^. Fixed-site measurements showed the worst performance, with the lowest ICC (0.39) and the largest bias (5.26 μg·m^–3^).

For BC (Figure 4), despite outdoor home measurements exhibiting a high ICC (0.73), its *B*_nmbf_ (0.29) and *E*_nmaef_ (0.39) both exceed the criteria showing a moderate level of agreement. Mobility-based estimates also resulted in a moderate agreement (ICC = 0.52), surpassing home-based estimates (ICC = 0.43) by about 21%. Fixed-site measurements had the lowest ICC (0.26). The sensitivity test using light absorbance measurements as the benchmark shows similar results (Supporting Figure S5 and Table S2). Mobility-based estimates still have better agreement with the benchmark than home-based estimates.

### 3.3 Stratified Analysis

Supporting Table S3 summarizes the results of the stratified analysis. The agreements between all methods and personal measurements of PM_2.5_ and BC were higher in participants with <1 h in-transit than those with ≥1 h. The agreement between personal measurements and mobility-based estimates was consistently larger than that with home-based estimates. Participants with less in-transit time showed an excellent agreement (ICC = 0.77) between mobility-based estimates and personal measurements of PM_2.5_ but moderate for BC (ICC = 0.63). Population with longer in-transit time exhibited a moderate agreement for PM_2.5_ and a poor agreement for BC between mobility-based estimates and personal measurements.

### 3.4 Assessment of Adjustment Factors

We computed ICCs between personal measurements and three models of mobility-based estimates with incremental adjustment factors (Table 2). Model 1 with only temporal adjustment still showed higher levels of agreement with personal measurements than home-based estimates (PM_2.5_: 0.41 versus 0.39, BC: 0.45 versus 0.43). After correcting for temporal and microenvironmental adjustment factors in Model 2, we observed large increases in the agreement with personal measurements of 56% for PM_2.5_ and 16% for BC. However, the ICCs of Model 2 (Table 2) and the fully adjusted Model 3 (Figures 3 and 4) were nearly identical, with *B*_nmbf_ and *E*_nmaef_ also closely aligned.

**Table 2:** Agreement Statistics on Exposure to PM_2.5_ and Black Carbon (BC) between Personal Measurements and Mobility-Based Estimate Models (i.e., Models 1, 2, and 3) Adjusted for Different Factors^a^^,^^b^^,^^c^^,^^d^

	mobility-based estimates	ICC (95%CI)	*B*_nmbf_ (***E***_nmaef_)
PM_2.5_	model 1	0.41 (0.24, 0.55)	0.46 (0.64)
	model 2	0.64 (0.56, 0.75)	0.05 (0.30)
	model 3	0.64 (0.54, 0.74)	0.06 (0.31)
BC	model 1	0.45 (0.27, 0.56)	0.43 (0.58)
	model 2	0.52 (0.37, 0.65)	0.24 (0.46)
	model 3	0.52 (0.40, 0.66)	0.27 (0.47)

a ICC = Intraclass correlation coefficient; *B*_nmbf_ (*E*_nmaef_) **=** Normalized mean bias factor (Normalized mean absolute error factor).

b Model 1 = LUR model + temporal adjustment factor.

c Model 2 = LUR model + temporal + microenvironmental adjustment factors.

d Model 3 = LUR model + temporal + microenvironmental + travel mode adjustment factors.

## 4 Discussion and Conclusions

This study is among the first to quantitatively evaluate mobility-based estimates’ agreement with personal measurements and its improvement in the agreement with personal measurements as compared to traditional home-based assessments using an older adult sample. We compared four typical exposure assessment methods with the benchmark assessed by personal measurements. Our results revealed that each method overestimated the actual exposure to air pollution (i.e., the benchmark) to some extent. Fixed-site measurements (from fixed monitoring sites) performed worst for both PM_2.5_ and BC. Mobility-based estimates notably improved the validity of the PM_2.5_ exposure assessment by 64% and of BC by 21% compared to home-based estimates. In our sample, the improvement in the agreement between mobility-based estimates and real exposure was primarily attributed to the microenvironmental adjustment factor distinguishing indoor-outdoor exposure concentration, with no added value observed from travel mode adjustments.

Our results showed that the assessed personal exposures were higher than the benchmark. Static methods (i.e., home-based estimates, outdoor home measurements, and fixed-site measurements) were found to have higher levels of exposure concentrations than dynamic methods (i.e., mobility-based estimates). As fixed-site measurements and the LUR model were based on different instruments, differences in level can be due to instruments. These findings were contrary to previous studies which have suggested that the daily exposure assessed by the mobility-based method is higher than the home-based method assessment.^27^ This contradiction may be due to the use of penetration factors in our study, which adjusts for indoor and outdoor air pollution differences in our sample. Given the fact that our older participants spent, on average, 19 h at home, the cumulative indoor/outdoor exposure difference can be profound. Our results align with previous findings that the fixed-site measurements are a poor proxy for personal exposure.^50^ Though fixed-site monitors usually have extensive coverage, they fail to capture hyperlocal variation in air pollution due to a sparse geographic distribution of the monitoring sites.^51,52^ Moreover, outdoor home measurements showed better agreement with the benchmark than home-based estimates. However, it is worth noting that deploying gravimetric samplers at participants’ homes incurs heavy costs and workload. Therefore, the potential improvement in the validity of exposure assessment due to outdoor home measurements is less likely to outweigh the associated costs, particularly in large-population studies. Advances in low-cost sensors may change this in the future.

Congruent with prior studies, our findings suggested that mobility-based estimates are valid to simulate personal exposures.^16,21,24,26,50,53^ We found that mobility-based estimates were relatively more valid than home-based estimates, which conflicted with earlier results.^24,50^ Those studies reported that personal measurements have stronger correlations with home-based estimates than mobility-based estimates.^24,50^ The discrepancy in the study population could explain different findings, but it also implies that the suitable exposure assessment method for specific population groups may vary. Moreover, PM_2.5_ showed a greater improvement (64%) in the agreement with actual exposure than that of BC (21%). A possible explanation is the larger variation of BC concentration across different microenvironments (e.g., home, dining, and shopping) compared to PM_2.5_^54^ Given that we only accounted for indoor versus outdoor air pollution concentration with a simple penetration factor, the adjustments for BC could be insufficient. In addition, some previous works suggested to use home-based estimates as a proxy for mobility-based estimates of PM_2.5_ because of the high correlation between exposure concentrations estimated by these two methods.^13,55^ We also observed that home-based estimates were highly correlated with mobility-based estimates; thus, we believe that home-based estimates could serve as an alternative to mobility-based estimates for PM_2.5_ when GPS data or time-activity recordings are unavailable. However, there were striking discrepancies in their agreements with the benchmark, and the assessed exposure concentration levels. Such a difference may translate into a change in estimated effect sizes when linking the exposure to health outcomes.

Our results indicated that the improvement in the agreement between mobility-based estimates and the benchmark stems from the microenvironmental adjustment. When focusing on participants with shorter in-transit time, mobility-based estimates showed significant improvements in the agreement with real exposure, with a 20% improvement for PM_2.5_ and 21% for BC. This result aligns with earlier findings that incorporating microenvironments results in large variations in modeled personal exposure concentration.^24,50,54^ More importantly, Lin et al.^54^ observed higher PM_2.5_ and BC concentrations in cycling and bus environments. These two travel modes contribute to ∼ a third of seniors’ travels in a study in Rotterdam.^30^ Therefore, the significance of travel mode adjustment should be stressed, as our results indicated. For participants with an in-transit duration of ≥1 h, the performance of the mobility-based estimates in assessment accuracy significantly decreased. This fact entails that the travel mode-adjusted mobility-based exposure deviated substantially from the real exposure. A possible explanation could be that these adjustment factors were compiled from several European studies^45^ and thus may not be perfectly suitable for Amsterdam and Utrecht. It could also relate to different environment settings within the same travel mode which could also alter pollutant concentrations.^45,56,57^ For instance, differences in ventilation conditions inside a vehicle can lead to changed pollutant concentrations.^57^ Lastly, it could well be that the validity of the exposure surfaces for PM_2.5_ and BC are not accurate enough on this hyperlocal scale to allow such detailed and nuanced linkage of position and time on an individual level.

Though our study confirmed better validity of the mobility-based method in an older adult population, it did not support replacing home-based estimates with mobility-based estimates in all circumstances. Incorporating more detailed and precise data (e.g., mobility data) may produce more accurate personal exposure estimates, but it comes with higher costs and higher participation burden. The key to the balance of the costs and gains is to what extent exposure assessments can be improved. In practice, when only GPS data and air pollution maps are available, home-based estimates are still recommended. The differences in ICCs between Model 1 and home-based estimates in our study are minor. Considering that home-based exposure assessments typically involve more participants at a lower cost than mobility-based assessments, a small improvement in terms of validity does not justify the use of mobility-based estimates. However, if additional data (e.g., microenvironment adjustment factors) are also available, our results support the use of mobility-based over home-based estimates as the improvement in exposure assessment is significant. However, we need to stress that our results originated from the analysis of older people and transferring these findings to the general population must be done with care.

To have a better understanding of the use of exposure assessment methods, several implications are suggested for future studies. First, it is advocated to explore if LUR-based air pollution maps can contribute to more improvements in the validity of exposure assessment. For example, air pollution maps at finer temporal scales (e.g., hourly) may result in more accurate personal exposure estimates than using yearly averages. Second, it is of particular interest to investigate to what extent an improvement in the validity of exposure assessment can translate into significant improvements in estimated effect sizes on health. This would shed light on how to determine the balance between costs and gains in mobility-based exposure assessments. Third, mobility-based estimates used in our study can be improved further. The microenvironment adjustment could go beyond simply distinguishing between indoor and outdoor environments. Penetration rates between indoor and outdoor particles differ by the type or quality of dwellings. For example, houses using mechanical ventilation systems with air filters have lower penetration rates than those not.^42^ Consequently, varying penetration ratios would benefit the conversion of the indoor/outdoor particle concentrations in mobility-based estimates. However, these improvements would necessitate additional data (e.g., characteristics of dwellings) for personal exposure assessments, and obtaining or measuring these data may encounter challenges. Especially for ethical issues, ensuring the anonymity of participants, secure storage of GPS data, and avoiding misuse and unauthorized access are concerns.^58,59^ Besides, geo-locational privacy should be carefully protected in the research and publication, as spatiotemporal movements are highly personalized, to avoid spatial reidentification of individuals.^59^

This study had several limitations. First, we used a relatively small sample of older people. Given that young adults likely visit more activity locations throughout the day and travel more actively than older adults,^30^ our study might underestimate the performance of applying mobility-based estimates to the general population. In addition, our participants were not equally sized in different sex groups. Given the discrepant mobility patterns between males and females,^60^ the validity of the mobility-based exposure assessment method could vary by sex. To generalize our findings, we thus recommend replications in other population groups. Second, the LUR model-based air pollution maps used in this study predate GPS tracking. Though we used a time series of air pollutant measurements from the Dutch National Air Quality Monitoring Network for temporal exposure adjustment, the network may not accurately characterize the actual air pollution concentration across cities.^52^ However, the Netherlands can be regarded as one airshed, and temporal patterns between fixed-site measurement stations are high. As such, we regard the temporal adjustment that we did as sufficient. Third, personal measurements are affected by housing characteristics and indoor sources of PM_2.5_ and BC. Personal air pollution exposure is affected by outdoor and indoor sources, which makes it difficult to only capture personal exposure to ambient air pollution. There is, therefore, a discussion on whether personal measurement is a gold standard. However, given participants did not smoke at home, which is one of the major indoor sources, and we only monitored for 24 h each time, we think the influence of indoor sources is reduced. Fourth, measurement errors could influence the comparison of the methods. Different measurement methods and instruments were used to assess air pollution on various occasions in this study, and measurement errors are inevitable, possibly affecting the comparisons between methods. However, these measurement methods are calibrated and validated elsewhere;^36,37,61^ thus, the influence is likely minor. Fifth, participants were monitored for only 3 days. Short-term monitoring raises the possibility that participants were engaged in nondaily routine activities. However, this possibility is attenuated because our participants were randomly assigned to three different days across different seasons. Replication of our findings using long-term monitoring is urged. Sixth, personal and outdoor home measurements were performed with the same instrumentation, but fixed-site measurements and the LUR model were based on different instruments. Thus, differences in the absolute level in comparisons of different methods with personal measurements can be partly due to different instruments. The ICC is likely affected less than the bias. The comparisons in Figures 3 and 4 between personal measurements with outdoor home measurements and fixed-site measurements suggest that differences in level due to instrument were modest.

In conclusion, we used several statistical analyses that allow the quantification of the validity of typical air pollution assessment methods using an older adult sample. Our results suggest an improvement in the validity of estimating personal exposure to air pollution using a mobility-based approach over a home-based assessment, by 64% for PM_2.5_ and 21% for BC. Adjusting indoor-outdoor air pollution concentration differences improves the validity of the mobility-based approach by 56% for PM_2.5_ and 16% for BC than those not, while no added value was observed from adjustments for travel modes. The mobility-based assessment is an easy and relatively accurate exposure assessment approach for participants and researchers since participants typically carry smartphones throughout the day. Since most European countries currently give free access to the governmental air pollution monitoring network and some LUR-modeled air pollution maps are publically available (e.g., ELAPSE project; http://www.elapseproject.eu/), the mobility-based method is easy to implement for other studies and is not affected by anecdotic exposures, unlike direct dynamic methods. Consequently, mobility-based estimates offer a way forward for obtaining more accurate person-centric estimates of ambient air pollution exposure.

## Supporting Information Available

Locations of fixed-site monitoring stations; photos of the gravimetric sampler and MicroAeth monitor; median temporal adjustment factors with the first and third quantiles of PM_2.5_ and BC in 24 h for home-based and mobility-based estimates; median and interquartile range of assessed PM_2.5_ and BC concentrations; Spearman correlation matrix of PM_2.5_ and BC exposure levels across different methods; distribution of assessed BC concentrations across different exposure assessment methods; agreement statistics on exposure to BC between light absorbance measurements and other methods; and agreement statistics on exposure to PM_2.5_ and BC between personal measurements and other methods stratified on participants’ in-transit time (PDF)
