# Supplementary material for: Validity of Mobility-Based Exposure Assessment of Air Pollution: A Comparative Analysis with Home-Based Exposure Assessment
Source: Environ Sci Technol. 2024 Jun 5;58(24):10685–95. doi: 10.1021/acs.est.3c10867 (PMC11191597; doi:10.1021/acs.est.3c10867)
Supplement: Supplementary file 1 — es3c10867_si_001.pdf [file es3c10867_si_001.pdf]

Supplementary Information for:

Validity of mobility-based exposure assessment of air pollution: A comparative analysis with home-based exposure assessment

Lai Wei [a‡*], David Donaire-Gonzalez [b‡], Marco Helbich [a], Erik van Nunen [b], Gerard Hoek [b], Roel C.H. Vermeulen [b,c]

[a] *Department of Human Geography and Spatial Planning, Utrecht University, 3584 CB Utrecht, the Netherlands*

[b] *Institute for Risk Assessment Sciences, Utrecht University, 3584 CK Utrecht, the Netherlands*

[c] *Julius Centre for Health Sciences and Primary Care, University Medical Centre, 3584 CK Utrecht University, Utrecht, the Netherlands*

* Corresponding author (Email: l.wei@uu.nl)

‡ LW and DDG contributed equally to this work.

Summary: 8 pages, 3 tables, 5 figures

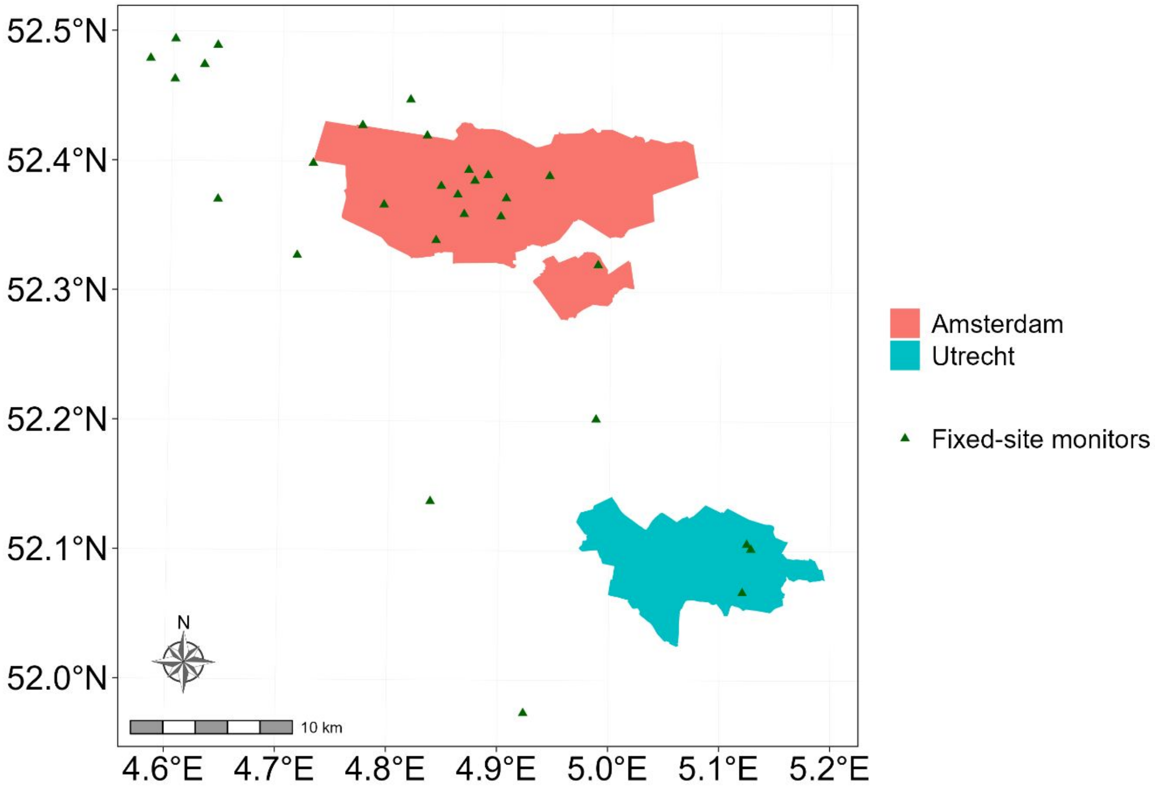

Figure S1. Locations of fixed-site monitoring stations near to Amsterdam and Utrecht.

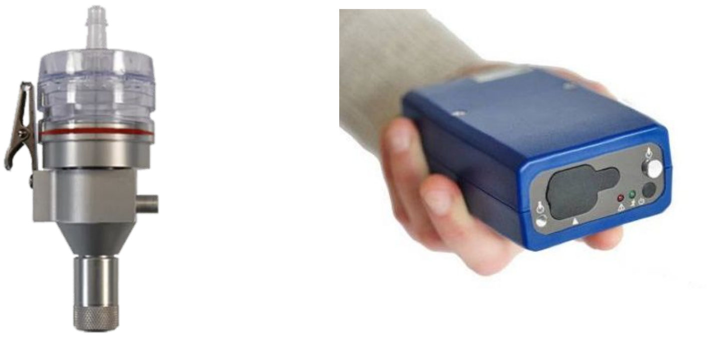

(a) (b)

Figure S2. Photos of (a) the gravimetric sampler (model GK2.05 SH, BGI Inc., Waltham MA, USA; image source: https://inteccon.com/products/bgi-cyclone-gk2-05-for-pm2-5-at-4-lpm/), and (b) the MicroAeth monitor (model AE51, AethLabs, San Francisco, CA; image source: https://app.swapcard.com/event/wwem-and-aqe-2022/product/UHJvZHVjdF83MjMzNDQ=)

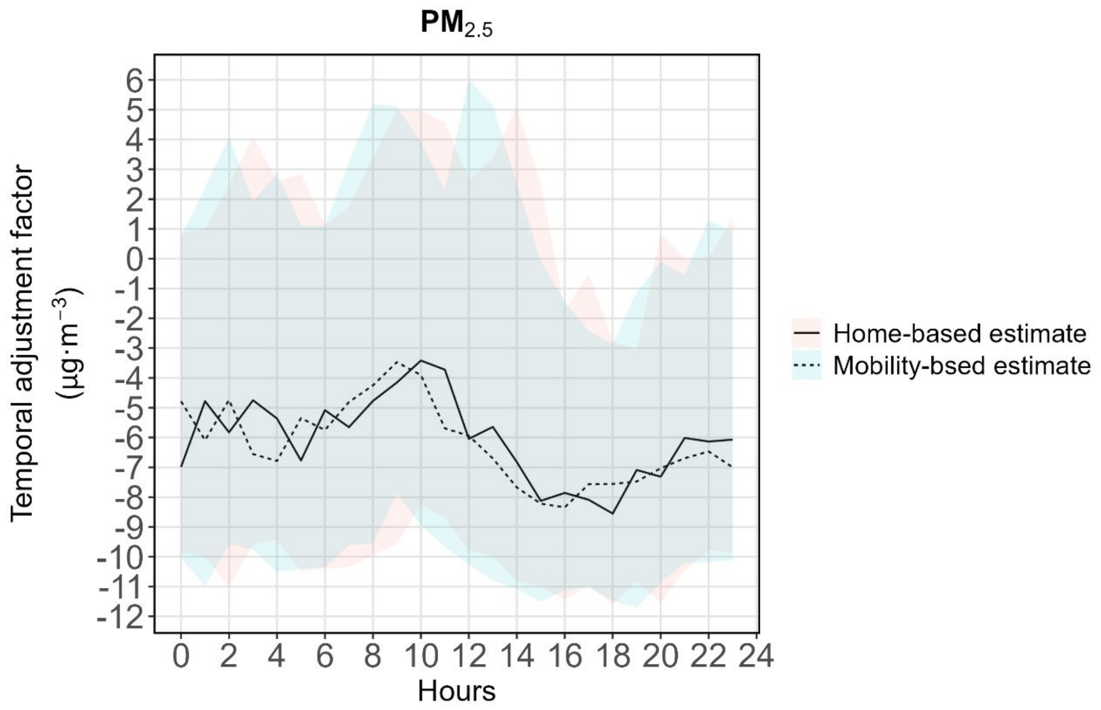

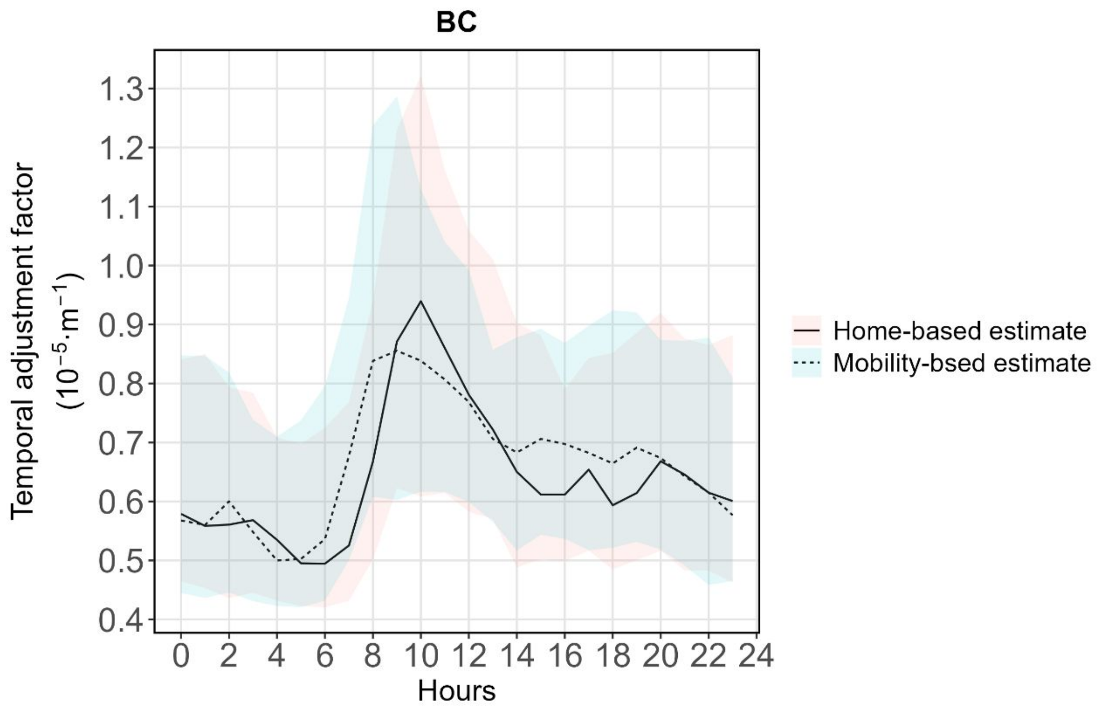

Figure S3. Median temporal adjustment factors with the first (the lower boundary of the shaded area) and third (the upper boundary of the shaded area) quantiles of $PM_{2.5}$ and BC in 24 hours for home-based and the mobility-based estimates.

Table S1. Median and interquartile range (IQR) of assessed $PM_{2.5}$ and BC concentrations. Note that BC from fixed-site measurements is in the unit of $\mu g \cdot m^{-3}$; both BC and $PM_{2.5}$ from fixed-site measurements were measured from different instruments than outdoor home and personal measurements.

	**$PM_{2.5}$ ($\mu g \cdot m^{-3}$)**		**BC ($10^{-5} \cdot m^{-1}$)**	
	**Median**	**IQR**	**Median**	**IQR**
Air pollution estimates				
Home-based estimates	12.36	10.82	1.23	0.60
Mobility-based estimates	9.64	5.13	1.10	0.47
Air pollution measurements				
Fixed-site measurements	12.66	9.64	1.35	0.63
Outdoor home measurements	10.24	9.57	0.99	0.89
Personal measurements	8.92	6.94	0.82	0.71

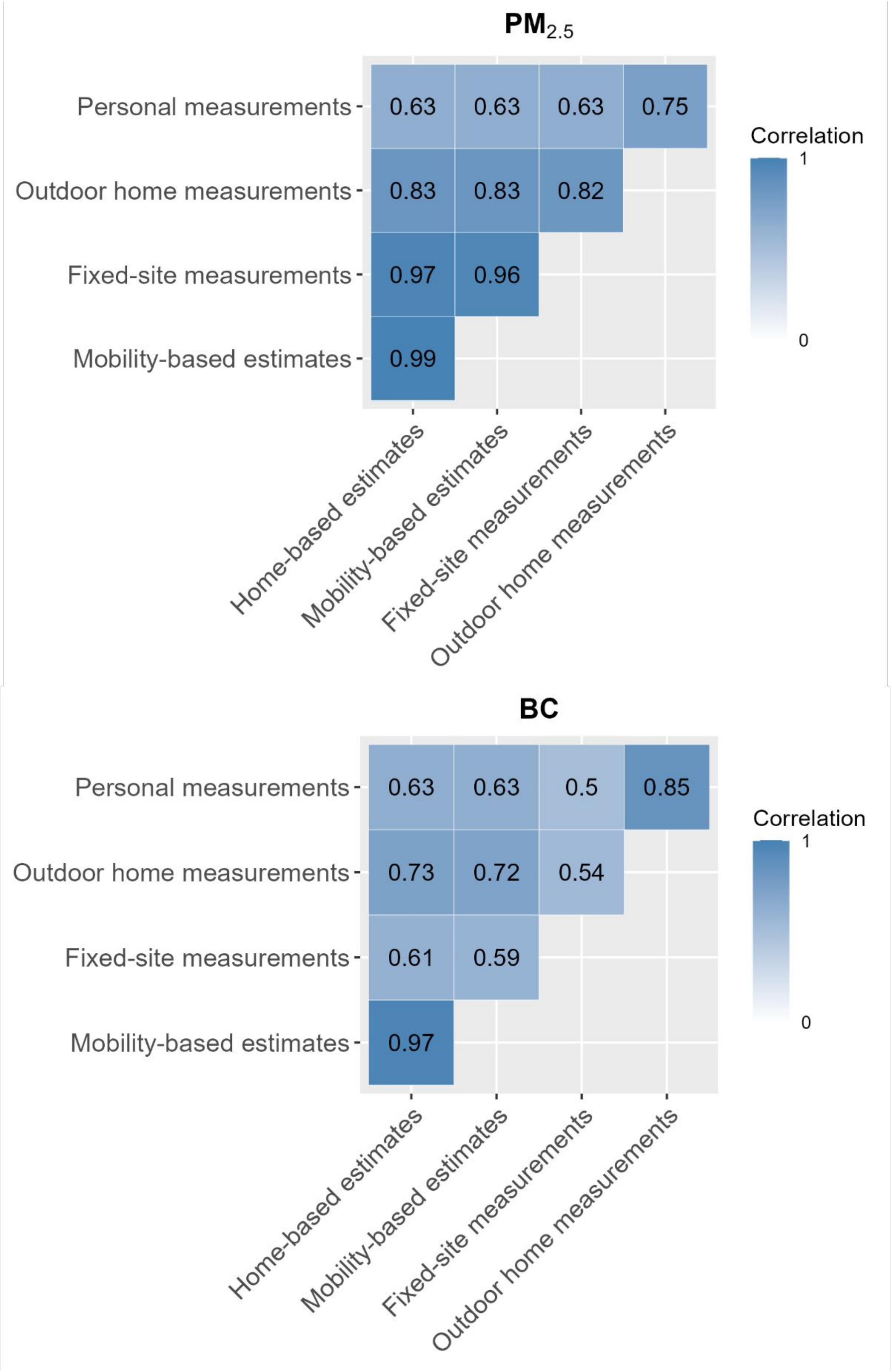

Figure S4. Spearman correlation matrix of $PM_{2.5}$ and BC exposure levels across different methods.

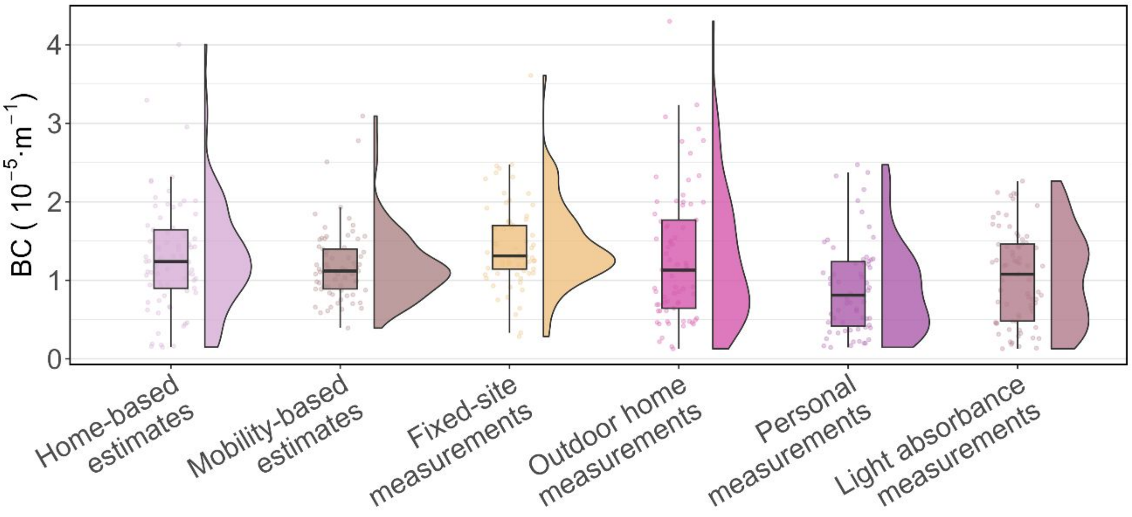

Figure S5. Distribution of assessed black carbon (BC) concentrations across different exposure assessment methods (N* = 73). Mobility-based estimates refer to Model 3 which were adjusted for temporal, micro-environmental, and travel mode factors. Note that BC from fixed-site measurements is in the unit of $\mu g \cdot m^{-3}$; both BC and $PM_{2.5}$ from fixed-site measurements were measured from different instruments than outdoor home and personal measurements. * Due to the mismatch of valid samples between personal measurements and light absorbance measurements, there are only 63 samples for personal measurements in this sensitivity test.

Table S2. Agreement statistics on exposure to BC between light absorbance measurements and other methods (*N** = 73). * Due to the mismatch of valid samples between personal measurements and light absorbance measurements, there are only 63 samples for personal measurements in this sensitivity test.

	**BC**	
	**ICC (95%CI)**	**B_{nmbf} (E_{nmaef})**
Air pollution estimates		
Home-based estimates	0.55 (0.36, 0.69)	0.25 (0.45)
Mobility-based estimates	0.58 (0.41, 0.70)	0.14 (0.38)
Air pollution measurements		
Fixed-site measurements	0.29 (0.07, 0.52)	0.38 (0.53)
Outdoor home measurements	0.67 (0.54, 0.78)	0.23 (0.40)
Personal measurements	0.86 (0.80, 0.92)	-0.16 (0.27)

Table S3. Agreement statistics on exposure to $PM_{2.5}$ and BC between personal measurements and other methods - stratified on participants' in-transit time.

	**$PM_{2.5}$**		**BC**	
	**ICC (95%CI)**	**B_{nmbf} (E_{nmaef})**	**ICC (95%CI)**	**B_{nmbf} (E_{nmaef})**
**In-transit < 1 hour** (*N = 54*)				
Air pollution estimates				
Home-based estimates	0.51 (0.26, 0.68)	0.45 (0.61)	0.60 (0.45, 0.73)	0.29 (0.57)
Mobility-based estimates	0.77 (0.63, 0.86)	0.00 (0.25)	0.63 (0.49, 0.75)	0.16 (0.43)
Air pollution measurements				
Fixed-site measurements	0.49 (0.18, 0.63)	0.47 (0.59)	0.42 (0.15, 0.60)	0.53 (0.64)
Outdoor home measurements	0.67 (0.49, 0.79)	0.26 (0.43)	0.81 (0.74, 0.88)	0.27 (0.34)
**In-transit ≥ 1 hour** (*N* = 50)				
Air pollution estimates				
Home-based estimates	0.27 (0.04, 0.53)	0.55 (0.73)	0.20 (0.03, 0.43)	0.50 (0.68)
Mobility-based estimates	0.50 (0.30, 0.68)	0.13 (0.38)	0.34 (0.08, 0.57)	0.41 (0.51)
Air pollution measurements				
Fixed-site measurements	0.28 (0.05, 0.50)	0.55 (0.71)	0.08 (0.01, 0.40)	0.81 (0.83)
Outdoor home measurements	0.48 (0.27, 0.67)	0.27 (0.52)	0.54 (0.27, 0.69)	0.30 (0.44)
